# Orthopedic Diseases in the Pura Raza Española Horse: The Prevalence and Genetic Parameters of Angular Hoof Deviations

**DOI:** 10.3390/ani13223471

**Published:** 2023-11-10

**Authors:** María Ripollés-Lobo, Davinia Isabel Perdomo-González, Pedro Javier Azor, Mercedes Valera

**Affiliations:** 1 Departamento de Agronomía, Escuela Técnica Superior de Ingeniería Agronómica, Universidad de Sevilla, 41013 Seville, Spain; marriplob@alum.us.es (M.R.-L.); dperdomo@us.es (D.I.P.-G.); 2 Real Asociación Nacional de Criadores de Caballos de Pura Raza Española, 41014 Seville, Spain

**Keywords:** equine, forelimbs, genetic parameters, heritability, pigeon-toed forelimb, pigeon-toed rear limb, splay-footed forelimb, splay-footed rear limb

## Abstract

**Simple Summary:**

This study attempts to elucidate the prevalence and genetic factors associated with four angular hoof deviations in the Pura Raza Española horse: splay-footed forelimb (SFF), pigeon-toed forelimb (PTF), splay-footed rear limb (SFR), and pigeon-toed rear limb (PTR). From a total of 51,134 horses evaluated, only 15.75% did not have any of the four investigated angular hoof defects, while the rest presented varying degrees of defect. We identified the factors influencing these defects, including age, inbreeding coefficient, sex, and birth stud size, using a Bayesian multivariate animal model with the BLUPF90 software. The heritability estimates ranged from 0.11 (SFR) to 0.31 (PTR) for model A (three categories.), indicating the genetic influences of varying categories; and ranged from 0.11 (SFR) to 0.29 (PTR) for model B (two categories). Additionally, the study explored the genetic correlations between these defects and their relationship with the diameter of the superficial digital flexor tendon (SDFT) and the proportionality index (PI). The findings revealed that diameter of the SDFT is strongly correlated with inward-toe conditions (PTF and PTR), while PI is associated with outward defects (SFF and SFR). These results provide valuable insights into angular hoof deviations in PRE horses, shedding light on their prevalence, genetic factors, and associations with other anatomical features, which can have general implications for a horse’s health and performance.

**Abstract:**

Abnormalities in hoof shape are usually connected with limb conformation defects. The role of angular hoof deviations is important for longevity in sports competitions and is increasingly recognized as a factor associated with lameness in performance horses. In this paper, we measured the prevalence of four defects related to the angulation of the hoof in the Pura Raza Española horse (PRE): splay-footed forelimb (SFF), pigeon-toed forelimb (PTF), splay-footed rear limb (SFR), and pigeon-toed rear limb (PTR). A total of 51,134 animals were studied, of which only 15.75% did not have any of the four angular hoof defects investigated, while 26.61%, 23.76%, 79.53%, and 3.86% presented SFF, PTF, SFR, and PTR, respectively. Angular defects were evaluated using two different models; model A was a linear scale composed of three categories, where 0 corresponded to the absence of defects, 1 to a minor presence of the defect and 2 to the highest degree of the defect. Model B was composed of two categories, where 0 corresponded to the absence of defects and 1 to the presence of defects, joining classes 1 and 2. We measured the factors influencing the appearance of these defects: age, inbreeding coefficient, sex, and birth stud size. The heritability of each defect was also estimated using a multivariate animal model, using the Gibbsf90+ software from the BLUPF90 family, resulting in heritability estimates of 0.18 (s.d. = 0.009), 0.20 (s.d. = 0.010), 0.11 (s.d. = 0.009), and 0.31 (s.d. = 0.010) for SFF, PTF, SFR, and PTR defects, respectively, for model A, and 0.17 (s.d. = 0.008), 0.19 (s.d. = 0.009), 0.11 (s.d. = 0.009), and 0.29 (s.d. = 0.009) for SFF, PTF, SFR, and PTR defects, respectively, for model B. Finally, the genetic correlation between the diameter of the superficial digital flexor tendon (SDFT) and the proportionality index (PI) in relation to the higher or lower prevalence of the defects was analyzed. We concluded that diameter of SDFT development is strongly correlated with inward toe conditions (PTF, PTR; *P*_≠0_ ≥ 0.95), while PI is associated with outward toe defects (SFF, SFR; *P*_≠0_ ≥ 0.95).

## 1. Introduction

The hoof structure plays a critical role in locomotion, weight distribution, and shock absorption during movement in equines [1], and disorders at the hoof level are considered to be a potential risk factor of lameness [2]. Defects in equine limbs usually consist of anomalies or irregularities in the structure or functionality of the joints in the limbs, including the hoof, the hock (tarsal joint), and the stifle [3]. Orthopedic diseases are of pa- ramount importance in equine medicine, as they have a significant impact on a horse’s health, performance, and longevity.

Angular hoof deviations encompass a spectrum of abnormalities that involve the alignment and orientation of a horse’s hooves in relation to the limb axis. These deviations impact the biomechanics of the horse’s limbs and, consequently, influence the horse’s performance in various activities, including work and sports competitions, or simply its ability to move comfortably without pain. These areas’ defects are associated with a series of factors, such as injuries, inflammation, bio-mechanical imbalances, abnormal development during the horse’s growth, or even genetic factors. The main consequence is the effect on the horse’s ability to move correctly [4,5]. Throughout history, horse breeders have developed routine trimming and shoeing techniques aimed at improving hoof balance by addressing factors such as hoof angle, length, mediolateral equilibrium, sole thickness, and the way the hoof interacts with the ground [6] to improve the horse movement. But also, a substantial body of research exists about the ‘optimal’ conformation of sport breed horses, such as Thoroughbreds [7,8,9,10], Warmbloods [11,12], Hanoverian warmblood horses [13], Trotting [14], Arabian [15], New Zealand Standardbred [16], Iceandic [17], and Pura Raza Española (PRE) [18]. Wilson et al. [6] identified a significant relationship between movement asymmetry and foot conformation in horses. In the same way, Ducro et al. [11] described that uneven feet have a moderate negative genetic relationship with foot conformation grade and this, in turn, has a genetic relationship with sporting performance in Warmblood riding horses. In addition, Ducro et al. [19] pointed out that uneven feet are known to negatively impact the competitive longevity of sport horses; given the observed prevalence of uneven feet in sports disciplines, it can be inferred that this is an undesirable characteristic, especially in elite jumping. Oosterlinck et al. [20] described that, while mild toed-in deviations have been found to have no significant impact on soundness or performance [21], there is empirical evidence suggesting that severe toed-in conformation is associated with an elevated risk of injury and a shortened lifespan for equine athletes. Nevertheless, more objective information regarding the biomechanical effects of toed-in and toed-out conformation during locomotion is necessary, even when, in horsemanship belief, the equine conformation is associated with performance and durability, as described in Thoroughbreds [9]. Thus, angular hoof deviations represent a specific area of interest, especially when considering the health, performance, and longevity on sport competition horses.

In this context, although all the above-mentioned risks produce themselves financial losses and a lower quality of life for the horse, from a population management point of view, knowledge about the genetic implications is of great interest. The identification and study of limb defects are essential for implementing the appropriate selection measures to reduce their incidence and improve the health and athletic performance of the breed, especially when considering sport competition horses such as the Pura Raza Española (PRE) horse. The PRE is a native Spanish breed, one of the oldest in Europe and one of the most important at the international level. This breed has gained international recognition in the field of Classical Dressage, taking part annually in a significant number of competitions, both in Spain and worldwide [22]. In general, PRE horses that excel in dressage performances have a higher economic value compared to other animals of the same breed, as this is one of the main objectives of their breeding program, which aims to enhance not only conformation and dressage functionality, but also, most importantly, gait quality, which is a key factor in optimizing dressage performance [23].

This study aims to provide a more comprehensive understanding of the factors affecting hoof health in PRE horses, with potential implications for the breeding program, management practices, and overall well-being of this equine breed. In this study, the incidence of hoof defects in the PRE horse population was estimated and the potential influencing risk factors in the presentation of angular hoof deviation were analyzed. Additionally, genetic parameters related to these defects were examined to determine if there is a genetic basis contributing to their occurrence. Finally, the relationship between the presence of hoof defects and morphological variables, specifically the diameter of the superficial digital flexor tendon (SDFT) and the proportionality index (PI), were explored.

## 2. Materials and Methods

### 2.1. Database and Description Traits

**Figure 1:**
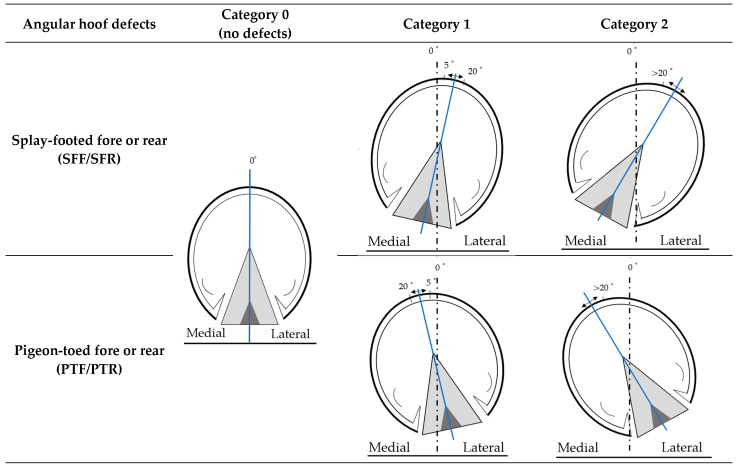
Conformational hoof defects (angular hoof deviations) in Pura Raza Española horses.

In our study, we analyzed hoof conformational data from 51,134 Pura Raza Española (PRE) horses (16,920 males and 34,214 females). The sample encompassed a variety of hoof conformational defects (Figure 1): hoof defects in the front legs were divided into ‘splay-footed forelimb’ (SFF) and ‘pigeon-toed forelimb’(PTF), and those in the back legs into ‘splay-footed rear limb’ (SFR) and ‘pigeon-toed rear limb’ (PTR). Therefore, each angular hoof defect was analyzed independently. The average evaluation age of the horses was 4.86 (s.d. = 2.25) years. Data collection took place between 2012 and 2022, during the mandatory morphological assessment, which occurs once in a horse’s lifetime before they are officially registered in the primary section of the PRE stud book. The evaluations were conducted by a total of 12 specially trained veterinarians responsible for performing standardized aptitude tests in this breed. Each horse was evaluated by a single evaluator. For the phenotypic evaluation of conformational defects, the horses were examined while standing on a firm, level surface with their limbs in a natural position. The fore and hind legs were placed in a parallel position as close to perpendicular as possible, with their hooves aligned. If the horse was still and there were doubts about the angular hoof defect, it was evaluated while the horse was in motion (walk or trot) to assign a defect category. For the evaluation of hoof deviation we considered, on the one hand, the forelegs, and, on the other hand, the hind legs for phenotyping angular defects in the limbs. This is because, in most cases, if one limb has an angular defect, it is common for the adjacent limb to also have one. Notably, no sedatives were administered during the evaluation process.

The four analyzed defects were as follows:

- Defects of the ‘splay-footed forelimb’ (SFF) and ‘pigeon-toed forelimb’ (PTF). These defects are related to the direction of the front hooves when seen from the front. An individual exhibits these defects when the hoof points either inwards or outwards, respectively, in relation to the vertical plumb line, as measured from the outer edge of the shoulder joint and the humero-cubital joint to the ground.
- Defects of the ‘splay-footed rear’ limb (SFR) and ‘pigeon-toed rear limb’ (PTR). These defects are related to the direction of the rear hooves when seen from the back. An individual has an SFR or PTR defect when the hoof tips either inwards or outwards, respectively, in relation to the vertical plumb line, as measured from the point of the hip and tibia-fibula joint to the ground, when seen from the rear.

The estimation of genetic parameters was performed with two different models:

For model A, the four conformational hoof defects were recorded on a linear scale from 0 to 2. Category 0: represents a lack of the defect, the hooves are vertical, or slightly turned outwards (SFF or SFR) or inwards (PTF or PTR), with, at most, 5 degrees of angulation. Category 1: a minor presence of the defect, the hooves are turned outwards (SFF or SFR) or inwards (PTF or PTR) between 5 and 20 degrees of angulation. Category 2: the most significant level of the defect, the hooves are turned outwards (SFF or SFR) or inwards (PTF or PTR) with more than 20 degrees of angulation.

For model B, the four conformational hoof defects were recorded as a dichotomous model. Category 0: represents a lack of the defect, the hooves are vertical, or slightly turned outwards (SFF or SFR) or inwards (PTF or PTR), with, at most, 5 degrees of angulation. Category 1: presence of the defect, the hooves are turned outwards (SFF or SFR) or inwards (PTF or PTR), with more than 5 degrees of angulation.

In PRE horses, SFF and SFR defects are considered to be severe when they are in category 2, and PTR and PTF defects are considered to be very severe when they are in category 2. When a PRE horse, in any part of its body, has more than 2 very severe defects or more than 4 severe defects, the horse cannot be registered as a breeder of the PRE breed.

The covariates analyzed were the evaluation age (in years; from 2 to 23 years) and the classical inbreeding coefficient (F; from 0.00 to 0.47). The F value, following Wright [24], defined as the probability that the two alleles at any locus in an individual are identical by descent, was computed using the Endog software (v.3.0) [25]. The fixed effects analyzed were: sex (2 levels; male and female), birth stud size (3 levels; small: <3 foals born/year, medium: from 3 to 9 foals born/year, and large: >9 foals born/year); those effects are statistically significative for the four angular hoof defects analyzed. A comparison of the different methodological approaches was based on the deviance information criterion (DIC) parameters [26].

Additionally, heritabilities and genetic correlations between the hoof defects and two morphological variables, the diameter of the superficial digital flexor tendon (SDFT), and the proportionality index (PI) were analyzed. The SDFT extends from the elbow region to the distal interphalangeal joint in the forelimb or from the hock region in the hind limb. The diameter of the SDFT was measured on a linear scale from 1 to 9, depending on the thickness of the tendon of the four limbs as a whole, where 1 is very thin and 9 is highly developed (larger diameter); the evaluation is unique, grouping, in a single evaluation, both forelimb and rear limb diameter. This tendon is a fundamental part of the connective system that allows for the function of hoof flexion in the horse’s limbs. The PI was defined as the height at the withers * 100 divided by the scapular-ischial length (distance of the straight segment from the union of the scapular-humerus joint to the point of the buttock). The PI is a variable widely used in equine breed characterization [27], and also because of its possible relationship with conformational defects [28].

### 2.2. Statistics and Genetic Analysis

A multivariate Generalized Non-linear Model (GLZ), with a multinomial logit distribution, was used to examine associations between the conformational hoof defects and their potential factors. All the statistical analyses were conducted using Statistica 11 for Windows software [29].

For the genetic evaluations, the genealogical information was obtained from the PRE StudBook managed by ANCCE [30], ensuring a minimum of 5 known generations for each animal in the performance control dataset. The pedigree file comprised total of 101,574 individuals. A multivariate model with the six variables (SFF, PTF, SFR, PTR, SDFT, and PI) was applied to study the genetic parameters of the conformational hoof defects. The equation in matrix notation used to solve the mixed model was:

**y** = **Xb** + **Zu** + **e**

$$u∼N(0,A\sigma_{u}^{2}),e∼N\left(0,\;I\sigma_{e}^{2}\right)\;$$

where **y** is the vector of observations, **X** is the incidence matrix of fixed effects, **Z** is the incidence matrix of the animal genetic effect, **b** is the vector of fixed effects, **u** is the vector of the direct animal genetic effect, **e** is the vector of residuals, $\sigma_{u}^{2}$ is the direct additive genetic variance, $\sigma_{e}^{2}$ is the residual variance, **I** is an identity matrix, and **A** is the numerator relationship matrix.

The genetic parameters were estimated using the Blupf90 software [31]. A Bayesian approach with threshold models was applied using GIBBSF90+. A total of 100,000 iterations were performed, with the initial 20,000 being considered as burn-in, after which, every 10th sample was saved. Therefore, 8000 chains were available for a post Gibbs analysis. Prior values were settled as similar to those reported for similar traits [3,32]. Finally, a post-Gibbs analysis was performed using POSTGIBBSf90 [33] to compute posterior means, additive and residual variances, and the standard deviations. Convergence was tested using the Z criterion of Geweke [34], and Monte Carlo sampling error was computed using time series procedures, as described in [35]. The Monte Carlo standard errors were small, ranging between 0.0004 (PTR) and 0.0010 (SFR) for model A, and ranging between 0.0004 (PTR) and 0.0020 (SFR) for model B. A lack of convergence was not detected by the Geweke test, ranging between −1.5130 (PTF) and 0.1670 (PTR) for model A, and ranging between −1.3929 (SFR) and 0.6989 (SFF) for model B. In order to identify the precision of the parameters, the 95% highest posterior density (HPD) intervals were determined from their marginal posterior distributions.

## 3. Results

The basic statistical results for angular hoof deviations in the PRE horses are shown in Table 1. The largest number of observations was for the SFR defect (50,717 records), with a mean value of 1.05, and the smallest number of observations was for the PTR defect (10,797 records), with a mean value of 0.05. Regarding the mode, all the defects had a mode of 1, except the SFR defect, with a mode of 0. Additionally, the highest 95% confidence interval was observed for PTF, and the smallest for PTR.

**Figure 2:**
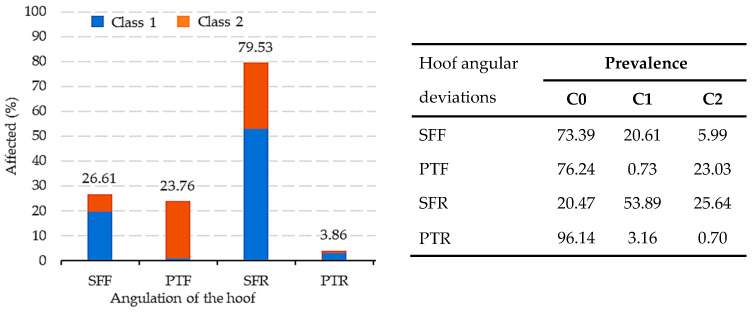
Percentage of affected animals for each of the angular hoof deviations in Pura Raza Española horse. SFF: splay-footed forelimb, PTF: pigeon-toed forelimb, SFR: splay-footed rear limb, and PTR: pigeon-toed rear limb.

The percentage of affected animals (category 1 or 2) for each hoof defect can be observed in Figure 2. For the SFF defect, category 0 had a prevalence of 73.39%, while categories 1 and 2 had a prevalence of 26.61% (20.61% and 5.99%, respectively, for categories 1 and 2). In the case of the PTF defect, category 0 had a prevalence of 76.24%, while categories 1 and 2 had a prevalence of 23.76% (0.73% and 23.03%, respectively, for categories 1 and 2). On the other hand, for the SFR defect, category 0 had a prevalence of 20.47% compared to a total of 79.53% of animals presenting this defect (53.89% and 25.64%, respectively, for categories 1 and 2). Category 0 for the PTR defect showed a prevalence of 96.14%, with only 3.86% of the animals presenting this defect (3.16% and 0.70%, respectively, for category 1 and 2). Finally, the percentages of PRE horses simultaneously exhibiting category 2 for the SFF-SFR, SFF-PTR, PTF-SFR, and PTF-PTR defects were 2.92%, 0.02%, 5.49%, and 0.05%, respectively.

In the analysis of angular hoof deviations in PRE horses, multiple effects were evaluated, including age, inbreeding, sex, and birth stud size (Table 2). A Generalized Non-Linear Model (GLZ) test revealed that, in the case of the PTF defect, all the effects studied were statistically significant. Regarding sex, significant differences were observed, with 20.34% of males and 25.30% of females being affected. Furthermore, birth stud size displayed statistically significant differences, with values of 23.90% (small), 23.46% (medium), and 23.99% (large) of animals being affected. In the case of the SFF and SFR defects, statistically significance differences were noted for age, but not for inbreeding. For SFF, statistically significant differences were found for sex, with 30.77% of males and 24.4% of females being affected. For SFR, significant differences were also found for birth stud size, with 78.44% of horses being affected in small studs, 79.92% in medium-sized studs, and 80.40% in large studs. Furthermore, for the PTR defect, no statistically significant differences were found for age or inbreeding, nor in sex. However, significant differences were observed for birth stud size, with 3.82% of animals being affected in small studs, 4.55% in medium studs, and 3.01% in large studs. These findings underscore the importance of considering age, inbreeding, sex, and birth stud size in genetic models when addressing angular hoof deviations and associated injuries in PRE horses.

**Figure 3:**
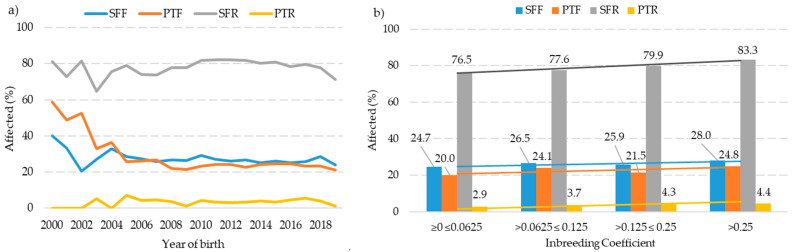
Evolution of the last 20 years of hoof defects in Pura Raza Española horses based on the year of birth (**a**) and incidence based on the level of consanguinity (**b**). SFF: splay-footed forelimb, PTF: pigeon-toed forelimb, SFR: splay-footed rear limb, and PTR: pigeon-toed rear limb.

The evolution of the four defects studied in the PRE horses over the last 20 years based on the horses’ birth year is shown in Figure 3a. In the first 5 years (from 2000 to 2005), irregularities were observed in the percentages affected for all four defects, with fluctuations during that period. From 2006, the percentages of animals affected stabilized. Notably, the SFR defect appeared to be the most prevalent, with an incidence ranging from 83% to 77% over time. Conversely, the PTR defect appeared to be the least prevalent, with an incidence fluctuating between 3% and 6% across the time series.

Figure 3b illustrates the incidence of the four hoof defects based on the inbreeding coefficient of the PRE horses. A clear upward trend in the number of individuals affected was observed as consanguinity rose. The SFR defect saw the most significant increase, going from 76.5% (in the range from ≥0 to ≤0.0625) to 83.3% (in the range of >0.25). On the other hand, the PTR defect exhibited a greater stability, ranging from 2.9% (in the range from ≥0 to ≤0.0625) to 4.4% (in the range of >0.25).

The variance components and heritability values for the four hoof defects studied in PRE horses are shown in Table 3. In model A, for the defects in the front hoof, heritability values of 0.18 (s.d. = 0.009) (SFF) and 0.20 (s.d. = 0.010) (PTF) were obtained, compared to values of 0.11 (s.d. = 0.009) (SFR) and 0.31 (s.d. = 0.010) (PTR) for back hoof defects. In model B, for the defects in the front hoof, heritability values of 0.17 (s.d. = 0.008) (SFF) and 0.19 (s.d. = 0.009) (PTF) were obtained, compared to values of 0.11 (s.d. = 0.009) (SFR) and 0.29 (s.d. = 0.009) (PTR) for back hoof defects. According to the DIC, the best fitting model was B. Although the genetic parameters obtained with model A and B were very similar, note that the percentage of animals that coincided between both models, according to the genetic value, in the 80th percentile (animals with higher genetic values for each of the defects), coincided at 96.67% for the PTR defect, at 86.65% for the PTF defect, at 84.98% for the SFF defect, and at only 20.50% for SFR defect.

The genetic correlations among the four hoof defects are presented in Table 4. In model A, the genetic correlation between PTF-SFR was negative, with a value of −0.31 (s.d. = 0.046), indicating an inverse association between these two defects. The other genetic correlations were positive, with a low to moderate magnitude, ranging between 0.09 (s.d. = 0.033) for SFF-PTR and 0.27 (s.d. = 0.031) for PTF-PTR. This genetic correlations were different from zero, as indicated by the symmetric HPD at 95% that did not include zero and the high probability to be greater or lesser than zero (*P*_≠0_ ≥ 0.95). On the other hand, for model B, the genetic correlations between SFF-PTR, SFF-SFR, and PTF-PTR were positive, with values of 0.14 (s.d. = 0.031), 0.35 (s.d. = 0.054) and 0.30 (s.d. = 0.030) for the three genetics correlations with a *P*_≠0_ = 1. The genetic correlation between PTF-SFR was small, with a value of −0.05 (s.d. = 0.055), and not different from zero, as indicated by the symmetric HPD at 95% that included zero (ranged from −0.053 to 0.159) and the low probability of being different from zero (*P*_≠0_ = 0.17).

Table 5 represents the means and heritability values for the diameter of the superficial digital flexor tendon (SDFT) and proportionality index (PI) in the dataset analyzed, as well as the genetic correlations between them and the four hoof defects, for both models, in the PRE horses. The means were 4.87 (s.d. = 1.083) for the SDFT and 99.80 (s.d. = 2.379) for the PI. For model A, the heritability of SDFT was 0.076 (s.d. = 0.014), and for PI, it was 0.348 (s.d.: 0.011), while for the model B, the heritability of SDFT was 0.077 (s.d. = 0.014), and for PI, it was 0.336 (s.d. = 0.010). Regarding the diameter of the superficial digital flexor tendon (SDFT), for model A, the PTF and PTR defect showed moderate, positive genetic correlations of 0.283 (s.d. = 0.060) and 0.276 (s.d. = 0.046), respectively. For model B, the PTF and PTR showed moderate, positive genetic correlations of 0.272 (s.d. = 0.051) and 0.266 (s.d. = 0.049), respectively. These genetic correlations were different from zero, as indicated by the symmetric HPD at 95% that did not include zero and the high probability to be greater than zero (*P*_≠0_ = 1.00) In model B, for the SFF and SFR defects, there was no evidence of a relationship between these factors (*P*_≠0_ = 0.77 and 0.03, respectively).

Regarding the proportionality index (PI), opposite correlations were observed compared to the SDFT. For model A, the defects SFF and SFR displayed low to moderate positive correlations of 0.212 (s.d. = 0.030) and 0.124 (s.d. = 0.041), respectively. For model B, the defect SFF displayed low to moderate positive correlation of 0.222 (s.d. = 0.031). In both models, for the PTF defect, there was no evidence of a relationship between these factors, not different from zero, as indicated by the symmetric HPD at 95% that included zero and the low probability to be lesser than zero (*P*_≠0_ = 0.87 in model A and *P*_≠0_ = 0.54 in model B). In model B, for the SFR defects, there was no evidence of a relationship between these factors, not different from zero, as indicated by its symmetric HPD at 95% that included zero and the probability to be lesser than zero (*P*_≠0_ = 0.94).

## 4. Discussion

Addressing horse limb defects, especially hoof angular deviations, is essential to ensuring a horse’s health, genetic quality, and performance, particularly in the context of sports competitions [36] and breeding programs. The uneven conformation of horses’ hooves is frequently studied and is of significant clinical importance for most equine breeds [37]. Angular deviation in a horse’s hoof leads to asymmetric limb loading, with subsequent implications for performance, injuries, and lameness [38]. Furthermore, Ducro et al. [19] demonstrated a link between uneven hoof conformation and a shorter competitive lifespan.

In the context of the basic statistical results, we observed significant variability in angular hoof deviations among the different defects analyzed (SFF, PTF, SFR, and PTR) in the PRE horses. The SFR defect exhibited the highest mean value and a wider confidence interval, indicating greater data dispersion compared to the PTR defect, which had the lowest mean value and the smallest confidence interval (Table 1). These intervals provide a measure of confidence in the variability of the mean estimates. Furthermore, this finding suggests the SFR defect was more prevalent compared to the PTR defect, as corroborated in Figure 2. The standard deviations turned out high, which is an indicator that further studies are needed to understand the mechanisms affecting the occurrence of these defects.

In the PRE horse, the outward deviation hoof defect can vary in severity and may appear in the forelimbs and/or rear limbs, although it is more common in the rear limbs (SFR) [39]. Horses, including racehorses, often exhibit some degree of inward-turning hocks [7], and as confirmed in the article by Ripolles et al. [3], in the case of PRE horses. This tendency can lead to the hoof turning slightly outward, contributing to a higher prevalence of SFR defect. The prevalence of this anomaly has been documented in various studies [40,41,42], underscoring this defect as one of the primary cases of angular deviation in the hindquarters of the equine breed. At times, the forelimbs may have good alignment, but are spoiled by a defect known as knock-knee. It can also occur that, while the metacarpal joint, carpal joint, and metatarsal bone are correctly aligned, the horse has SFF due to the outward twist of the fetlock, which usually causes the hoof to point in the same direction [43]. On the other hand, the inward deviation hoof defect can vary in severity, and can also appear in both pairs of limbs, although it is more common in the forelimbs (PTF). A very wide chest, conversely, results in widely separated front knee joints, leading to the hoof pointing inward [39]. In other cases, the defect exists when the horse has dropped heels, very long fetlocks, is bench-kneed, or has divergent hocks [43].

In each of the four hoof deviation defects, categories 1 and 2 represented the individuals affected, with significantly different percentages being observed among the defects (Figure 2). This indicates variations in the prevalence of the defects within the studied population. The prevalence of having any hoof deviation defect varies among breeds. In the PRE horse, it was 84.24%, which is similar to the prevalence in the Dutch Warmblood horse (85%) [44]. Moreover, this prevalence is lower in mules and Dutch Warmblood horses, with incidences of 37.1% [45] and 8% [11], respectively. It is important to focus on reducing the incidence because, as seen previously, horses that simultaneously exhibit PTR-PTF defects in category 2 (considering both defects are very severe for the PRE breed) are the animals most likely to be unsuitable as breeders for the breed, as they only require one more very severe defect, either in the limbs or elsewhere in the body, to be disqualified.

To achieve an accurate, comprehensive understanding of the patterns in hoof deviation defects, we need to consider that both environmental and genetic factors can influence the occurrence and severity of conformational defects. Although most widely published works have addressed the clinical perspective, no detailed genetic analyses for hoof deviation defects in PRE horses have been extensively conducted until now. In our study, we examined not only the influence of sex and age, but also the individual coefficient of inbreeding and the breeding stud size (Table 2). Overall, all these effects were statistically significant for most of the hoof deviation defects analyzed. The effects mentioned in this study have previously been implemented in estimations of the genetic parameters for morphological abnormalities in the PRE breed, such as cresty neck [46], ewe neck [28], or limb deformities [3], as well as those associated with medical conditions, like obesity [47] or the presence of cutaneous melanomas [48]. Additionally, they have also been included in the assessment of various PRE variables, including morphological [49] variables.

Hoof variations depend not only on the constant movements made during work [50] and the type of terrain where the horse treads, but also on the horse’s age. The age of evaluation plays a crucial role in the development and conformation of a horse’s limbs. Skeletal growth, hoof development, and the proper ossification of limb bones are critical factors to consider when evaluating hoof conformation. Newborn foals typically have a weaker conformation, narrow chests, and long legs relative to their size. This can lead to an outward hoof posture as they need to provide support to their forelimbs [51]. Over time, this may result in a correction of the inward hoof posture [52]. Events that affect a foal’s intrauterine environment, such as placentitis during pregnancy or severe metabolic diseases in the mare, can compromise the proper ossification of these bones, but this only becomes a problem when hoof conformation defects persist with age [51].

Regarding sex, males exhibited a higher incidence of hoof deviation defects compared to females, which aligns with the findings in the lameness study conducted by Ross [53]. This sex disparity may be linked to factors such as differences in the intensity of the use of the horse. In sports competitions, there is often a higher participation of males, and pregnant mares generally undergo less intense exercise.

An association between the size of the breeding stud and the percentage of affected horses was also observed. Large-scale birth studs primarily focus on breeding, while smaller and medium-sized ones are mainly dedicated to competition. This distinction is crucial, because the type of livestock management significantly influences aspects such as the type of exercise horses undergo, the management regimen, the intensity of training, and other related factors. In this context, it has been documented that foals raised in large herds with extensive management may develop limb conformation irregularities due to uneven load patterns during grazing [54], as horses tend to systematically extend the same limb while grazing [55]. While exercise is obviously crucial for development, excessive exercise can lead to the development of angular deviations [56]. Another relevant consi-deration is nutritional imbalance, which can influence disproportionate growth in foals.

Regarding the evolution of defects related to consanguinity levels (Figure 3b), it is evident that there is a trend toward an increase in the percentage of animals affected as inbreeding values rise. These results support the theory that inbreeding plays a significant role in the occurrence and transmission of these genetic defects in the horse population. Increases in consanguinity are typically associated with a decrease in animal fitness and, consequently, a reduction in their performance in terms of capacity or physiological efficiency [57]. This finding underscores the importance of carefully considering genetic diversity and relatedness in horse breeding programs.

Conformation defects have been evaluated in numerous equine breeds using a linear scale that usually ranges from 5 classes [32,58] or 9–10 classes [12,59,60,61], where opposing defects are placed in the classes located at the extremes of the linear scale (for example, in a 5-category scale, where category 1 would be the SFF defect at its maximum grade, category 2 the SFF defect at its low grade, category 3 the animal does not present any defect, category 4 would be the PTF defect at its low grade, and category 5 would be the PTF defect at its maximum grade). However, recently, Ripollés et al. [3] showed that the best methodology for the genetic evaluation of conformation defects is to use an independent scale for each of the defects, where all the scale categories refer to assessing the grades of the defect presentation considered. Probably some of the genes involved in the presentation of a particular defect, even if they are related to the same anatomical area, may be different. Another issue when using a very large scale to evaluate a defect, especially when it is complex to assess through visual inspection, such as angular hoof defects, is that eva-luators may not be able to utilize the entire scale and find it challenging to determine the differences between two consecutive categories. It is advisable to reduce the number of categories for phenotypically evaluating this type of defect. The heritabilities found in other studies have been higher than those obtained in the four defects studied in PRE horses (Table 3), with values of 0.45 in Icelandic horses [58], 0.40 in Brazilian sport horses [60], 0.12–0.27 found in KWPN breeds [11], and 0.26 (0.109) in the Pura Raza Menorquina [32]. The heritability of other hoof deformities was estimated to be 0.13 (0.022) for front hoof and 0.007 (0.018) for rear hoof in Warmblood foals [12] and 0.25 in Belgian horses [62]. In this study, despite the high residual variances, the heritabilities obtained suggest that selective breeding can be used to reduce the prevalence in the breed, even though the genetic component is smaller than the environmental one, with a moderate magnitude. It is also worth noting that the heritabilities from the two models (A and B) were very similar, so model B may also be effective when morphologically assessing angular hoof defects in horses. Although model B is the best fit, according to the DIC, it is important to work with three categories (model A), since, as has been seen, when we have a significant number of animals in category 1 and category 2 for a defect (example of the SFR defect; Figure 2), the percentage of animals that match according to the genetic value, at the 80th percentile, is very low (only 20.50% for the SFR defect). Therefore, model A allows us to diffe-rentiate the animals that transmit any of the defects studied to a greater degree. In this sense, it is very interesting for the breeder to be able to genetically identify the animals that are going to transmit some of the defects to a greater degree.

Regarding the genetic correlations between different hoof deviation defects, our results support that different sets of genes are involved in coordinating hoof deviation defects, depending on whether they occur in an outward or inward position (Table 4). In a study conducted in the Dutch Warmblood horse population, moderate correlations were observed between hoof deviation defects and traits of the distal limb [11]. Specifically, the heel height showed negative correlations with the cannon angle (−0.42) and hoof shape (−0.41), indicating that high heels are associated with narrow hooves and more vertical cannons.

The equine limb behaves like a large system of springs due to the extension of the metacarpophalangeal joint and the elastic recoil in tendons [63,64]; specifically, the SDFT is specialized in storing elastic energy [65]. It has been observed that the conformation of a horse’s hoof may be related to predisposition for certain tendon defects [66,67]. Angular hoof defects can influence how a horse distributes its weight when bearing weight, affec-ting the load on associated tendons and ligaments, thus increasing its susceptibility to injuries [36,68,69]. Injuries to the SDFT are common in racehorses, especially in the forelimbs, with a prevalence ranging from 10% to 30% [66,70]. Additionally, it has been observed that the prevalence of these injuries tends to increase with a horse’s age [71,72,73]. In our study, positive correlations were observed between the SDFT and angular hoof deviations (PTF and PTR; Table 5). These correlations may be related to the biomechanics of horse limbs. Excessive development of the SDFT could affect how a horse places its limbs during movements, which could influence their weight bearing and, in turn, contribute to certain inward hoof deviation defects. Additionally, Shade et al. [67] determined a correlation of −0.26 between static and dynamic angles of the metacarpophalangeal joint and the SDFT in Campeiro and Mangalarga Marchador horses.

On the other hand, the PI in horses seems to be a determining factor that influences movement and, consequently, plays an essential role in maintaining proper balance and hoof conformation [51]. This perspective underscores the importance of carefully consi-dering the relationship between PI and hoof angular deviations. Furthermore, the need for hoof volume to be correctly adjusted to the horse’s size and robustness, as suggested by Giles [74], fully justifies the inclusion of PI as a relevant study variable in this research. In contrast to SDFT, positive genetic correlations were observed between PI and outward angular deviations (SFF and SFR; Table 5), which suggests that horses with outward hoof defects tend to have a higher PI. Robust, broad-chested horses typically used for heavy pulling tasks have a lower withers height and often exhibit an inward hoof tread pattern, which is related to the occurrence of defects like PTF and PTR [1]. These types of horses tend to have larger hooves compared to saddle horses [51]. In contrast, horses destined for sports activities, such as dressage, as is the case with PREs, often exhibit a greater withers height and narrower chest conformation, resulting in a higher PI. These horses tend to bear weight in a way that directs the hooves outwards, which is associated with a more frequent occurrence of defects like SFF and SFR in breeds of this type. Furthermore, according to [75], there is a genetic correlation between height at withers and a hoof conformation of 0.15, which is directly related to PI.

It is possible that there may be limitations in this work, as it was not possible to stan-dardize the age of all the animals since the animals were not evaluated at the same age. Additionally, the presence of animals assessed both with and without horseshoes, as well as their participation in competitions, could have introduced biases in the assessment of angular defects, as footwear and exercise can impact the correction of these issues. Another limitation of phenotyping in the study is the assessment by a single evaluator, rather than using consensus from multiple evaluators for each horse, and variability between horses since there were different single evaluators across the large dataset. Despite this, this work had a significant representation of active PRE horses and it demonstrated that the angular hoof deviations affect limb biomechanics, influencing the horse’s ability to perform, which may also have genetic implications, justifying the implementation of sui-table selection measures to reduce their incidence and improve both the breed and equine sports performance. All these findings emphasize the importance of breeding programs that not only focus on the quality of movements and functionality in dressage, but also place a fundamental emphasis on conformational defects of the horses’ limbs.

## 5. Conclusions

A high prevalence of angular hoof defects was found in the PRE population, with a higher occurrence in males and significant variations with an increasing inbreeding coe-fficient and breeder herd size. It was demonstrated that genetic factors play a role in the occurrence of these defects, with moderate heritability values. Furthermore, we observed genetic correlations between the defects analyzed, pointing to the existence of specific genes related to the presence of particular defects. Our study also underscored the signi-ficance of taking into account factors such as the proportionality index (PI) and development of the superficial digital flexor tendon (SDFT). It revealed correlations between these factors and the direction of angular hoof defects, with the SDFT being associated with inward defects and the PI with outward defects. Breeders and veterinarians should take into account, when organizing the mating of the stud, the genetic values of the breeding animals for each of the angular hoof deviation defects (SFF, PTF, SFR, and PTR), along with the genetic values of the rest of the morphological defects of the breed, both in the limbs and other areas of the body. From a reproductive perspective, breeders should prioritize horses whose genetic values, for each of the conformation defects analyzed in the breed (regardless of the anatomical region), have a high probability of not being transmitted to the offspring. Well-organized horse pairings based on the genetic value of the offspring will reduce defects in the population.

## Figures and Tables

**Table 1:** Basic statistics of angular hoof defects in the Pura Raza Española horse.

Hoof Angular Deviations	N	Mean ^1^ (s.e.)	s.d.	Mode	Confidence ^2^ −95%	Confidence ^2^ +95%
SFF	41,614	0.326 (0.003)	0.583	0	0.320	0.332
PTF	40,061	0.467 (0.004)	0.842	0	0.460	0.476
SFR	50,717	1.051 (0.003)	0.677	1	1.046	1.058
PTR	10,797	0.045 (0.002)	0.240	0	0.041	0.050

^1^ Mean: mean value of angular deviation score based on 0–2 ratings. ^2^ Confidence intervals of the mean. N: number of records, s.e.: standard error, s.d.: standard deviation, SFF: splay-footed forelimb, PTF: pigeon-toed forelimb, SFR: splay-footed rear limb, and PTR: pigeon-toed rear limb.

**Table 2:** Generalized Non-linear Model (GLZ) between the angular hoof deviations with the risk factors for all PRE horses analyzed.

Hoof Angular Deviations	Age (*p*-Value)	Inbreeding Coefficient (*p*-Value)	Sex			Birth Stud Size			
			Male (%)	Female (%)	*p*-Value	Small (%)	Medium (%)	Large (%)	*p*-Value
SFF	0.050	0.477	30.77	24.40	<0.001	26.90	26.01	27.02	0.259
PTF	<0.001	0.019	20.34	25.30	<0.001	23.90	23.46	23.99	0.006
SFR	<0.001	0.568	79.64	79.40	<0.001	78.44	79.92	80.40	<0.001
PTR	0.829	0.652	4.21	3.69	0.301	3.82	4.55	3.01	0.007

SFF: splay-footed forelimb; PTF: pigeon-toed forelimb; SFR: splay-footed rear limb; PTR: pigeon-toed rear limb; small: <3 foals born/year; medium: 3 to 9 foals born/year; large: >9 foals born/year, and (%): percentage of animals affected.

**Table 3:** Genetic parameters of the angular hoof deviations studied in Pura Raza Española horses.

Model	Hoof Angular Deviations	σ_u_			σ_e_			b_inbreeding_ (s.d.)	b_age_ (s.d.)	h^2^ (s.d.)	DIC
		Mean	Median	HPD 95%	Mean	Median	HPD 95%				
A	SFF	246.57	246.80	218.40–269.90	1140.70	1141.00	1118.00–1166.00	9.90 (4.418)	−0.57 (0.079)	0.18 (0.009)	−360,278,759.93
	PTF	325.95	325.40	293.60–359.20	1274.40	1274.00	1246.00–1304.00	−3.72 (4.680)	0.45 (0.083)	0.20 (0.010)	
	SFR	12.02	11.98	10.22–13.98	94.72	94.72	92.88–96.67	−1.36 (1.213)	−0.07 (0.021)	0.11 (0.009)	
	PTR	444.55	444.70	410.90–475.00	1000.20	619.10	975.10–1026.00	7.75 (4.647)	0.40 (0.076)	0.31 (0.010)	
B	SFF	6.93	6.92	6.27–7.73	34.81	34.80	34.03–35.62	1.80 (0.754)	−0.09 (0.014)	0.17 (0.008)	−781,593,187.00
	PTF	8.63	8.64	7.82–9.52	36.32	36.22	35.64–37.20	−0.28 (0.838)	0.06 (0.014)	0.19 (0.009)	
	SFR	1.05	1.05	0.80–1.29	8.49	8.48	8.26–8.70	−0.23 (0.392)	0.01 (0.006)	0.11 (0.009)	
	PTR	14.22	14.21	13.19–15.34	33.89	33.89	33.23–34.62	1.45 (0.850)	0.07 (0.018)	0.29 (0.009)	

SFF: splay-footed forelimb; PTF: pigeon-toed forelimb; SFR: splay-footed rear limb; PTR: pigeon-toed rear limb; σ_u_: additive genetic variances; σ_e_: residual variances; HPD 95%: 95% higher posterior density; h^2^: heritabilities; s.d.: standard deviation; b: slope, and s.d.: standard deviation.

**Table 4:** Genetic correlations (*r_g_*) among the angular hoof deviations studied in Pura Raza Española horses.

Model		*r_g_* (s.d.) PTR	HPD 95%	*P*_≠0_	*r_g_* (s.d.) SFR	HPD 95%	*P*_≠0_
A	SFF	0.09 (0.033)	0.025–0.154	1.00	0.09 (0.045)	0.003–0.180	0.97
	PTF	0.27 (0.031)	0.207–0.331	1.00	−0.31 (0.046)	(−0.389)–(−0.208)	1.00
B	SFF	0.14 (0.031)	0.086–0.200	1.00	0.35 (0.054)	0.228–0.444	1.00
	PTF	0.30 (0.030)	0.251–0.364	1.00	−0.05 (0.055)	−0.053–0.159	0.17

SFF: splay-footed forelimb, PTF: pigeon-toed forelimb, SFR: splay-footed rear limb, PTR: pigeon-toed rear limb, *r**_g_***: genetic correlations, s.d.: standard deviation, and HPD 95%: 95% higher posterior density. *P*_≠0_ (*r_g_* > 0): probability of genetic correlation being higher than zero. *P*_≠0_ (*r_g_* < 0): probability of genetic correlation being lower than zero.

**Table 5:** Mean and heritability (h^2^) of the superficial digital flexor tendon development (SDFT) and proportionality index (IP) variables and their genetic correlations (*r_g_*) with angular hoof deviations in Pura Raza Española horses.

		SDFT			PI		
**Mean (s.d.)**		4.87 (1.083)			99.80 (2.379)		
**h^2^ (s.d.)**	Model A	0.076 (0.014)			0.348 (0.011)		
	Model B	0.077 (0.014)			0.336 (0.010)		
***r_g_*** **(s.d.)**			**HPD 95%**	***P*_≠0_**	***r_g_*** **(s.d.)**	**HPD 95%**	***P*_≠0_**
Model A	SFF	−0.081 (0.054)	(−0.188)–0.028	0.93	0.212 (0.030)	0.151–0.270	1.00
	PTF	0.283 (0.060)	0.171–0.400	1.00	−0.034 (0.030)	(−0.093)–0.024	0.87
	SFR	−0.113 (0.064)	(−0.234)–0.003	0.96	0.124 (0.041)	0.039–0.201	1.00
	PTR	0.276 (0.046)	0.188–0.367	1.00	−0.088 (0.026)	(−0.142)–(−0.039)	1.00
Model B	SFF	−0.040 (0.052)	(−0.145)–0.057	0.77	0.222 (0.031)	0.165–0.293	1.00
	PTF	0.272 (0.051)	0.167–0.364	1.00	0.037 (0.031)	(−0.046)–0.069	0.54
	SFR	−0.118 (0.068)	(−0.014)–0.243	0.03	0.071 (0.045)	(−0.012)–0.160	0.94
	PTR	0.266 (0.049)	0.171–0.357	1.00	−0.088 (0.025)	(−0.138)–(−0.035)	1.00

SDFT: superficial digital flexor tendon; PI: proportionality index; SFF: splay-footed forelimb; PTF: pigeon-toed forelimb; SFR: splay-footed rear limb; PTR: pigeon-toed rear limb; s.d.: standard deviation; and HPD 95%: 95% higher posterior density. Highest posterior density interval at 95% in brackets. *P*_≠0_ (*r_g_* > 0): probability of genetic correlation being higher than zero. *P*_≠0_ (*r_g_* < 0): probability of genetic correlation being lower than zero.

## Data Availability

The data used in this article is part of the performance control data carried out as part of the Pura Raza Español Breeding Program (BOE nº 175 of June 24, 2020. Sec. III Pág. 44191, https://www.mapa.gob.es/es/ganaderia/temas/zootecnia/ancceen_tcm30-540900.pdf, accessed on 1 June 2023), whose technical director of the Breeding Program is Dr. M. Valera (author of this work and director of Research Group PAIDI-AGR-273 the University of Seville, a qualified center for genetics https://servicio.mapa.gob.es/arca/flujos.html?_flowExecutionKey=c21e6192-8dd3-470b-8216-e0cc38ed413f_MTAzQjRDMDYxODMwNjgwQzIzNEI0RDc5NDBBMjAzODIuZTA0MTAwNjA=&_eventId=desplogarResponsa-ble&indiceResponsable=0, accessed on 1 June 2023).
